# Differences among Saudi and Expatriate Students: Body Composition Indices, Sitting Time Associated with Media Use and Physical Activity Pattern

**DOI:** 10.3390/ijerph17030832

**Published:** 2020-01-29

**Authors:** Ahmad H. Alghadir, Zaheen A. Iqbal, Sami A. Gabr

**Affiliations:** 1 Rehabilitation Research Chair, College of Applied Medical Sciences, King Saud University, Riyadh 11433, Saudi Arabia; 2 Department of Anatomy, Faculty of Medicine, Mansoura University, Mansoura 35516, Egypt

**Keywords:** Saudi Arabia, expatriates, students, physical activity

## Abstract

*Background*: Being overweight at a young age is a predictor of developing obesity and related complications later in adulthood, posing a high risk to public health. Various ethnic subgroups have been identified as having a higher prevalence of overweight or obese. Saudi Arabia is one of the fastest-growing economies in the world, where the expatriate population comprises 33% of its total population. The objective of this study was to investigate differences in body composition indices, sitting time associated with media use, and physical activity pattern among a sample of local and expatriate school students in Saudi Arabia. *Methods*: 500 students (aged 8–18 years) from various schools were invited to participate in this study. Body weight, waist circumference (WC) and height were measured using a portable digital metric scale, standard measuring tape and wall mounted tape respectively. Participants and their parents were jointly asked to report the average time that the participant spent sitting using media (watching TV, playing video games, and using the internet and other screen-based devices etc.) per day. The pattern of physical activity among participants was measured using a short form of the International Physical Activity Questionnaire (IPAQ). Basal metabolic rate (BMR) and total daily energy expenditure (TEE) were estimated from body weight, height, age, sex and physical activity, according to the Harris–Benedict equation. *Results*: Data from 450 (90%) of the participants were used for analysis. The mean age of the participants was 14.55 ± 1.74 years. Body mass index (BMI), WC, waist to height ratio (WHtR), BMR and TEE differed significantly among the participants. Physical fitness score negatively correlated with BMI and WC, while sitting time associated with media use positively correlated with BMI, WC, WHtR and physical fitness score, among both Saudi and expatriate participants. *Conclusions*: Body composition indices and sitting time associated with media use were higher among Saudi boys and expatriate girls. Expatriate boys and girls were reported to be physically more active than their Saudi counterparts. BMR and TEE were higher among expatriate boys and Saudi girls. Although this study provides useful information about the association of body composition indices, sitting time associated with media use, and physical activity pattern among local and expatriate school students in SA, similar studies involving a larger study sample, with equal gender representation, are further required to determine various factors associated with this link.

## 1. Introduction

Overweight and obesity pose a high risk to public health [1,2,3]. Being overweight at a young age is a predictor of the development of obesity and related complications later in adulthood [4,5]. During adolescence, health-related behaviors like a sedentary lifestyle, low physical activity pattern and energy expenditure are subject to change, which may lead to the development of unfavorable health patterns in adulthood [6,7].

The time children spend doing physical activities like athletics, football, etc., has been overtaken by sitting time associated with media use, including watching television (TV), playing video games, and using the internet and other screen-based devices [8,9,10]. Leisure time has become more sedentary because of the easy availability of the internet on mobile phones, providing access to TV and video games all the time [11]. A previous study has shown that young adults spend almost twice as much of their leisure time using computers compared to older adults [12]. This has been attributed to the availability of TV and computers in the child’s bedroom [13,14]. Such sedentary activities, when associated with low intensity physical activity and increased intake of high-calorie food and drinks, have been collectively shown to cause weight gain among young adults [15,16,17]. A WHO review report has identified that eating meals while food advertisements are aired on TV has a direct effect on child eating behavior, and encourages food preferences that pose high risks for the development of obesity [18,19,20].

The effect of watching TV on the body weight of children has been shown to differ from country to country, depending on traditions and culture associated with TV content [13,21]. Various studies have shown that although White students spend less time watching TV, they showed strong associations between TV watching time and being overweight, sedentary, and eating unhealthy foods, compared to Black and Hispanic youth [22,23,24]. Other studies with nationally representative samples have also reported results consistent with these findings [25,26].

Besides body mass index (BMI), physical activity has been shown to improve cognitive and academic performance in individuals of all ages [27]. A decrease in physical activity and an increase in body weight has been shown to occur at the same time, especially in school children [28,29]. In children, low physical activity has been shown to affect social and environmental changes that initiate sedentary behaviors [30].

There are various studies that report a high prevalence of overweight, obesity and related complications among the Saudi population [31], especially in children and adolescents [32,33]. There are studies that have associated this with the rise in sedentary behavior, lack of physical activity, and local eating habits in the country, including consumption of high-calorie foods and beverages [8,14,28]. Saudi Arabia (SA) is one of the fastest-growing economies in the world [34]. Various skilled and unskilled workforces from countries like India, Pakistan, Bangladesh, Egypt, the Philippines, Afghanistan and other African countries are known to migrate there. Most of them are with their families, in search of a better life, and meeting local work requirements [35]. According to the Saudi government’s Central Department of Statistics and Information, 33% of the total population consisted of foreigners in 2014, equivalent to 10.1 million in numbers. They are commonly referred to as expatriates [36]. To the best of our knowledge, despite this high number there are no studies that have investigated overweight, obesity and related factors among these expatriates, or compared it with the local population.

Various ethnic subgroups and people of lower socio-economic status have also been identified as having increased prevalence rates of overweight and obesity [37,38,39]. This has been connected to demographic variation, as well as cultural and lifestyle differences in underlying causes of weight gain, which is further affected by many factors including differences in energy intake and expenditure [22,40]. Therefore, there is a need to examine the relationship between ethnicity and levels of physical activity [41].

This study was done to compare expatriate children with Saudi children and see if cultural differences affect their energy-related behavior. The objective of this study was to investigate the differences in body composition indices, sitting time associated with media use, and physical activity pattern among a sample of local and expatriate school students in Saudi Arabia.

## 2. Materials and Methods

A total of 500 expatriate and Saudi students (aged 8–18 years) from several schools in SA were invited to participate in this study. Firstly, school principals were briefed about the need and importance of the study. Once necessary permission was obtained, they connected us with the students and their parents. Participants were classified as Saudi if both parents were born in SA, and as an expatriate if both parents were born outside SA. They were excluded if either of the parents was Saudi or born outside the SA. Participants reporting any kind of disability were excluded from the study. All the participants and their parents had to provide informed consent to participate in the study. This study was approved by the ethical committee of the institutional review board.

Body weight was measured using a portable digital metric scale that was calibrated using standard weights. Height was measured using a wall-mounted tape. These values were used to calculate their BMI. Waist circumference (WC) was measured as the minimum circumference between the iliac crest and the rib cage [42]. The waist-to-height ratio (WHtR) was calculated by dividing the waist by the height [43,44]. Sitting time associated with media use was calculated as TV viewing and computer usage times (hours per day). TV viewing time was defined as the time spent watching TV or DVDs, while computer usage time was defined as the time spent on a home computer, laptop, mobile phone, or playing videogames. All the information was jointly provided by parents and participants. They were asked to report average time participants spent sitting using media (watching TV, playing video games, and using internet and other screen-based devices etc.) per day [45].

The pattern of physical activity among participants was measured using the short form of the International Physical Activity Questionnaire (IPAQ) [46,47,48]. They were asked to report the average time per week that the participant spent on physical activity. The amount (in minutes) per week of walking and moderate and vigorous physical activity was computed according to the IPAQ scoring manual [49]. The participants were classified into mild (≤500 METs-min/week), moderate (500–2500 METs-min/week) and active (≥2500 METs-min/week) groups according to their physical activity level. The basal metabolic rate (BMR) and total daily energy expenditure (TEE) were estimated from body weight, height, age, sex and physical activity according to the Harris–Benedict equation [50].

Statistical Analysis

Data were presented as mean ± standard deviation (SD). Statistical analysis was performed using SPSS version 13.0 for Windows (SPSS Inc., Chicago, IL, USA). Firstly, the normality of the data distribution was assessed using the Kolmogorov–Smirnov test, and since body mass values were skewed, a non-parametric (Mann–Whitney U) test was used to compare the data. Association between BMI, physical activity level and other variables was calculated using correlation coefficients (r and β) as tests. Statistical significance was accepted for values of *p* ≤ 0.05.

## 3. Results

Out of 500 invited students, 475 provided the completed written informed consent form. Of these, complete measurements and data were obtained from 460 participants. However, after applying the exclusion criteria, data from 450 (90%) of the participants were used for analysis. Mean age of the participants was 14.55 ± 1.74 years. Saudi people and expatriates (225 of each) were equally distributed among participants. In the Saudi group, there were 125 boys and 100 girls. In the expatriate group, there were 115 boys and 110 girls. Among expatriate participants, 90 were Egyptian, 50 were Indian, 45 were Pakistani, 25 were Afghan, 10 were South African, and 5 were Australian. Demographic and anthropometric data of the participants based on gender and nationality are presented in Table 1.

### 3.1. Body Composition Indices

Data on body composition indices are presented in detail in Table 1. BMI, WC and WHtR differed significantly among the participants. Among boys, BMI, WC and WHtR were higher among Saudi participants (*p* ≤ 0.05). Among girls, BMI, WC and WHtR were higher among expatriate participants (*p* ≤ 0.05).

### 3.2. Sitting Time Associated with Media Use

There were significant differences in sitting time associated with media use in both the groups (*p* ≤ 0.05). Saudi boys spent more time watching TV and using computers than expatriate boys (Table 2). On the other hand, expatriate girls were shown to use the TV and computer more than Saudi girls (Table 3).

### 3.3. Physical Activity Pattern

Based on physical activity score, participants were classified under mild, moderate and active groups (Table 2 and Table 3). Overall, 48% of each the Saudi (n = 60) and expatriate (n = 55) boys were in the active group. On the other hand, 45% (n = 45) of Saudi girls and 23% (n = 25) of expatriate girls were found to be in the active group. Although a greater number of Saudi boys reported to be active, expatriates spent more time in physical activities in all the three groups, and reported to have higher energy expenditure (*p* ≤ 0.05). Among girls, despite having low energy expenditure, expatriates were more active than their Saudi counterparts (*p* ≤ 0.05).

### 3.4. Relation between Body Composition Indices, Sitting Time Associated with Media Use, and Physical Activity Pattern

Physical fitness score negatively correlated with BMI, WC, WHtR, and TV/Computer use time, while it positively related with TEE among both Saudi and expatriate participants (*p* ≤ 0.05) (Table 4). However, sitting time associated with media use (TV and computer) correlated positively with BMI, WC, WHtR and physical fitness score among all participants (*p* ≤ 0.05) (Table 5 and Table 6).

## 4. Discussion

To the best of our knowledge, this is the first study in the region to compare local and expatriate school students on differences in body composition indices, sitting time associated with media use, and physical activity pattern. The results show that body composition indices and sitting time associated with media use were higher among Saudi boys and expatriate girls. Expatriate boys and girls reported to be physically more active than their Saudi counterparts. BMR and TEE were higher among expatriate boys and Saudi girls. Physical fitness score negatively correlated with BMI and WC, while sitting time associated with media use positively correlated with BMI, WC, WHtR and physical fitness score among both Saudi and expatriate participants.

Previous studies have reported ethnic behavioral differences related to energy expenditure during TV watching, physical activity and food intake [1,14,51,52]. This has been attributed to interaction of biological and social factors with an environment that allows less physical activity due to weather and over consumption of high-calorie food and drink [14,33,53]. Although our findings are consistent with previous studies suggesting the association of time spent watching TV, using computers, and doing physical activity with higher BMI [54], our results show no consistent difference pattern among Saudi and expatriate girls and boys. One of the reasons could be that a large part of the expatriate population in SA constitutes people from Egypt, also represented in our sample, who have a similar culture and eating habits.

Besides genetics, nutrition, and hormones, ethnicity and level of physical activity one performs are major determinants of normal body growth and maturation in children. These factors can also affect various other biological traits and body composition [55,56]. Most of the participants in this study were adolescents. Adolescent age is associated with stress that can lead to over- or under-eating [57]. There are various studies that have associated emotional symptoms, stress in life, life satisfaction, food insecurity, family stressors, dietary practices, and physical activity buffers with obesity and weight gain, in culturally diverse adolescents [57,58]. In SA, favorable socioeconomic factors, cultural factors, access and availability of food, education and age have been reported to affect the choice of what local people eat especially in children and adolescents, and this can affect their health [59].

Partial correlation and regression analysis showed significant positive correlation between BMI, WC, WHtR and physical fitness score and duration of TV viewing and computer usage time, among both Saudi and expatriate participants. This result supports previous studies showing that greater TV viewing time is associated with higher BMI [60,61], especially in children [21]. Although there was a positive correlation between TEE and physical fitness score, our results also show that TEE was higher in expatriate boys and Saudi girls compared to their counterparts. This may be the reason that in spite of greater time spent doing sedentary activities, BMI is higher among expatriate girls and Saudi boys.

Our study also examined physical activity levels in Saudi and expatriate participants. The participants were classified into mild (55 Saudis, 65 expatriates), moderate (65 Saudis, 80 expatriates) and active (105 Saudis, 80 expatriates) groups. Despite a greater number of Saudi boys reporting to be active, expatriates spent the most time doing physical activities out of all three groups. Expatriates were more active than all other girls also. The physical fitness score negatively correlated with sitting time associated with media use, and physical activity negatively correlated with body composition indices. This is in agreement with similar findings reported by numerous studies, which have proposed that besides limiting TV watching time [62,63], inclusion of daily physical activity is an essential intervention to prevent obesity among schoolchildren [8,27,64]. This will help not only reduce energy intake, but increase its consumption also [65]. It has been shown that even a small difference in physical activity can affect body composition [1], and the type of physical activity done also makes a difference. Owing to the favorable climate and higher price of transport fuel, it is a common practice in western countries to walk or cycle to school [66]. This is not common in SA due to hot weather, traffic and cheaper fuel prices. Active transport to school (walking or cycling) is a great way to promote physical activity among students and should be promoted to keep them fit.

Although expatriate girls reported to be physically more active, they also reported to watch TV and use computers more than Saudi girls and all the boys. Females have been shown to have longer average TV and computer usage time in other studies as well [67,68]. Girls have been shown to have greater body weight and lower height in comparison to boys across the world [69,70].They also have differences in muscle strength, muscle composition, hematological parameters and ventricular chamber sizes. Some literature shows a gender-based disparity in physical activity among youth, where girls are less active than boys [71]. This difference lowers their physical fitness, since their body reacts differently to the reduced physical activity and increased sedentary activities. Moreover, SA is a country with a relatively closed culture, where there is a high level of segregation between men and women at public places like parks, gyms, etc., where ladies can go for jogging or exercise. The impact of such differences in the region needs to be studied further in order to prevent and improve negative consequences of overweight and related complications in the future.

### Limitations

This study used self-reported data from participants and their parents. There is a possibility that they may have over or underestimated their experiences. There was a difference in the distribution of participants between the groups of Saudi and expatriate participants, based on gender and individual nationality. The places where participants were born, the duration for which they have been in SA and their individual nationality were not considered during analysis. More similar studies, with larger samples and an equal gender distribution are required to further determine the factors associated with such a link. They can also help to develop targeted, effective and culturally appropriate interventions for all from different cultural backgrounds.

## 5. Conclusions

This study shows that body composition indices and sitting time associated with media use were higher among Saudi boys and expatriate girls. Expatriate boys and girls reported to be physically more active than their Saudi counterparts. BMR and TEE were higher among expatriate boys and Saudi girls. Physical fitness score negatively correlated with BMI and WC, while sitting time associated with media use positively correlated with BMI, WC, WHtR and physical fitness score among both Saudi and expatriate participants.

## Figures and Tables

**Table 1:** Demographic and anthropometric data of participants: Mean ± SD (n = 450).

	Boys			Girls		
Characteristics	Saudi (n = 125)	Expatriate (n = 115)	*p*-Value	Saudi (n = 100)	Expatriate (n = 110)	*p*-Value
**Age (year)**	15.0 ± 1.84	14.5 ± 1.65	0.29	14.8 ± 1.77	13.9 ± 1.72	0.11
**Height (cm)**	169.2 ± 7.5	168.3 ± 3.1	0.158	166.3 ± 7.16	171.3 ± 6.4	0.187
**Weight (kg)**	68.76 ± 7.5	67.5 ± 6.1	0.125	65.9 ± 8.8	68.3 ± 9.6	0.129
**BMI**	23.9 ± 4.4	23.7 ± 1.74	0.001	24.74 ± 3.0	25.4 ± 3.6	0.01
**WC (cm)**	95.3 ± 12.0	93.5 ± 13.8	0.01	98.7 ± 12.2	102.4 ± 12.8	0.001
**WHtR**	0.57 ± 0.09	0.56 ± 0.08	0.001	0.57 ± 0.11	0.59 ± 0.21	0.05

Data expressed as mean ± SD. WC: Waist Circumferences (cm); WHtR: Waist to height ratio.

**Table 2:** Sitting time associated with media use and Physical activity pattern among Saudi and Expatriate boys.

	Saudi (n = 125)	Expatriate (n = 115)	*p*-Value
**Media use** **(h/d)**			
Television Viewing	2.92 ± 1.84	2.74 ± 1.13	0.05
Computer usage	2.79 ± 0.87	2.77 ± 0.84	0.01
**Physical activity** (min/week)			
Mild	356 ± 48.7 (n = 25)	485 ± 25.5 (n = 20)	0.001
Moderate	1875 ± 120 (n = 40)	1980 ± 65 (n = 40)	0.001
Active	3955 ± 250 (n = 60)	4690 ± 85.7 (n = 55)	0.001
**Energy expenditure rates** (kcal/day)			
BMR	1742 ± 142.3	1988 ± 148.9	0.001
TEE	2099 ± 449.45	2102 ± 454.7	0.003

Data expressed as mean ± SD. BMR: basal metabolic rate (kcal/day); TEE: total energy expenditure (kcal/day).

**Table 3:** Sitting time associated with media use and Physical activity pattern among Saudi and Expatriate girls.

	Saudi (n = 100)	Expatriate (n = 110)	*p*-Value
**Media use (h/d)**			
Television viewing	2.8 ± 1.24	3.5 ± 1.3	0.01
Computer usage	2.68 ± 0.91	2.8 ± 0.98	0.05
**Physical activity** (min/week)			
Mild	248 ± 21.6 (n = 30)	328 ± 45.2 (n = 45)	0.001
Moderate	1645 ± 56.3 (n = 25)	2180 ± 75 (n = 40)	0.001
Active	2685 ± 175 (n = 45)	3190 ± 85.7 (n = 25)	0.001
**Energy expenditure rates** (kcal/day)			
BMR	1507 ± 100.2	1186 ± 115.2	0.05
TEE	2210 ± 426.03	2178 ± 327	0.02

Data expressed as mean ± SD. BMR: basal metabolic rate (kcal/day); TEE: total energy expenditure (kcal/day).

**Table 4:** Correlation between body composition indices, energy expenditure, media usage and physical fitness score among participants using partial correlation and multiple regression analysis.

Body Composition Indices, TEE Rate and Media Usage	Physical Fitness Score			
	Saudi		Expatriate	
	R	β	r	β
**BMI**	−0.216 **	−0.025 **	−0.123 **	−0.182 **
**WC (cm)**	−0.635 **	−0.038 **	−0.965 **	−0.045 **
**WHtR**	−0.480 **	−0.018 **	−0.325 **	−0.035 **
**TEE (kcal/day)**	0.315 **	0.68 **	0.158 **	0.78 **
**TV/Computer use time (h/d)**	−0.125 ***	−0.98 **	−0.250 ***	−0.72 **

** *p* < 0.01; *** *p* < 0.001. BMR: basal metabolic rate (kcal/day); TEE: total energy expenditure (kcal/day); BMI = Body mass index, WC = Waist circumference, WHtR = Waist height ratio.

**Table 5:** Correlation between body composition indices, physical fitness score and media usage among participants using partial correlation and multiple regression analysis.

Body Composition Indices and Physical Fitness Score	TV (h/d)		Computer Usage (h/d)		
	r	r	β	r	β
**BMI**	0.25 **	0.45 **	0.23 **	0.74 **	0.32 **
**WC (cm)**	0.13 **	0.64 **	0.58 **	0.25 **	0.32 **
**WHtR**	0.141 **	0.149 **	0.121 **	0.152 **	0.221 **
**Physical fitness score**	0.11 **	0.15 **	0.27 **	0.32 **	0.29 **

** *p* < 0.01; BMI = Body mass index, WC = Waist circumference, WHtR = Waist height ratio.

**Table 6:** Correlation between body composition indices, physical fitness score and media usage among participants using partial correlation and multiple regression analysis.

Body Composition Indices and Physical Fitness Score	TV (h/d)		Computer Usage (h/d)	
	r	β	r	β
**BMI**	0.085 *	0.073 *	0.034 *	0.012 *
**WC (cm)**	0.37 *	0.29 *	0.39 *	0. 51 *
**WHtR**	0.129 **	0.167 **	0.147 **	0.137 **
**Physical fitness score**	0.19 *	0.21 *	0.27 *	0.31 *

* *p* < 0.05, ** *p* < 0.01. BMI = Body mass index, WC = Waist circumference, WHtR = Waist height ratio.

## Data Availability

The datasets used in this study are available from the corresponding author on request.
